# Temporally replicated DNA methylation patterns in great tit using reduced representation bisulfite sequencing

**DOI:** 10.1038/s41597-019-0136-0

**Published:** 2019-07-24

**Authors:** Hannu Mäkinen, Heidi M. Viitaniemi, Marcel E. Visser, Irene Verhagen, Kees van Oers, Arild Husby

**Affiliations:** 1 Organismal and Evolutionary Biology Research Programme, University of Helsinki, Helsinki, Finland; 2 Department of Animal Ecology, Netherlands Institute of Ecology (NIOO-KNAW), Wageningen, The Netherlands; 3 Evolutionary Biology, Department of Ecology and Genetics, Uppsala University, Uppsala, Sweden; 4 Centre for Biodiversity Dynamics, Department of Biology, NTNU, Trondheim, Norway

**Keywords:** DNA sequencing, Epigenomics, DNA methylation

## Abstract

Seasonal timing of reproduction is an important fitness trait in many plants and animals but the underlying molecular mechanism for this trait is poorly known. DNA methylation is known to affect timing of reproduction in various organisms and is therefore a potential mechanism also in birds. Here we describe genome wide data aiming to detect temporal changes in methylation in relation to timing of breeding using artificial selection lines of great tits (*Parus major*) exposed to contrasting temperature treatments. Methylation levels of DNA extracted from erythrocytes were examined using reduced representation bisulfite sequencing (RRBS). In total, we obtained sequencing data from 63 libraries over four different time points from 16 birds with on average 20 million quality filtered reads per library. These data describe individual level temporal variation in DNA methylation throughout the breeding season under experimental temperature regimes and provides a resource for future studies investigating the role of temporal changes in DNA methylation in timing of reproduction.

## Abstract (summary)

**Table:** 

Design Type(s)	DNA methylation profiling by high throughput sequencing design • time series design • parallel group design
Measurement Type(s)	DNA methylation
Technology Type(s)	reduced representation bisulfite sequencing assay
Factor Type(s)	experimental condition
Sample Characteristic(s)	Parus major • blood • laboratory environment

Machine-accessible metadata file describing the reported data (ISA-Tab format)

## Background and Summary

In seasonally varying environments, timing of reproduction is under strong selection, as individuals need to adjust the time of reproduction to favorable environmental conditions. Individuals that are reproducing too early or too late in relation to the peak in food abundance may have reduced fitness^1,2^. Understanding how organisms translate environmental cues into phenotypes, such as timing of breeding is therefore important for predicting how individuals and populations respond to changing environmental conditions. A well-known environmental cue that plants and animals use to time their reproduction is photoperiod^3,4^. However, additional cues are also involved and increases in yearly temperatures has led to changes in seasonal timing of breeding in many plants and animals^5^. For example, in some passerine birds, the timing of reproduction has advanced about 0.25 days per year during the last three decades^6,7^. The shift towards earlier breeding in many species in the Northern hemisphere is likely an adaptive response to the increase in temperature and the resulting shift in the timing of emergence of their prey^1,8^.

While we have a quite good understanding of the environmental factors and selective agents operating on seasonal timing of reproduction in birds, our knowledge about its genetic basis is poor. Seasonal timing of reproduction is frequently found to be heritable^9–12^ but the underlying genes involved and how environmental cues are sensed and translated into a physiological response remains largely unknown.

Recently, several studies have indicated DNA methylation as a potential mechanism that may modulate gene expression via environmental stimuli^3,13^. Due to technical advances in next generation sequencing the characterization of DNA methylation has become a popular tool, also in non-model organisms^14^. The identification of methylated sites is based on bisulfite conversion of un-methylated C’s to T’s but methylated C’s are not affected. In most methylation analyses sequencing reads are mapped to a reference genome and those sites showing no C to T change are considered as methylated sites. Furthermore, techniques such as reduced representation bisulfite sequencing (RRBS) that use a restriction enzyme (such as MSPI in most vertebrates) enriching for CpG rich regions, allow for cost efficient methylation profiling^15^.

The great tit has become an ecological model species for understanding the impact of climate change on many different aspects, including morphological changes^16^, population sizes^17^ and reproductive related traits^18,19^. Earlier studies have found that great tits adjust their timing of breeding based on local environmental conditions^9,10^, indicating that this trait is phenotypically plastic^9–11^. Experimental studies have demonstrated that temperature is causally related to the initiation of timing of breeding in great tits^20–22^. Thus, temperature may result in differences in methylation profiles for individuals exposed to different temperatures causing gene expression differences among these individuals, leading to differences in timing of reproduction. While there are a number of ecological studies examining DNA methylation it is often difficult to rule out potential confounding factors behind observed methylation changes in natural populations. To avoid this problem, we used great tit blood samples from birds originating from a genomic selection experiment for early timing of breeding that were kept in climate-controlled aviaries^23^. This allowed us to control for variation in factors such as food availability and age of the birds that could otherwise confound our results. We characterized DNA methylation patterns using reduced representation bisulfite sequencing (RRBS) of females subjected to either a cold or a warm temperature treatment. We sampled each individual in a temporally replicated manner during the breeding season to better understand potential seasonal variation in DNA methylation as well as in relation to experimental temperature effects.

## Methods

### Samples for sequencing

We used samples from great tit females belonging to a large-scale artificial selection experiment (for more exact details see^23^). In short, DNA samples of erythrocyte origin, from offspring (first generation ‘F1’) from phenotypically early and late wild breeding pairs (parental generation ‘P’) were collected and genotyped using a 650k SNP chip^23^. Genomic breeding values (GEBVs) were calculated, which were used for bi-directional genomic selection for early or late reproduction. GEBV is the value of an individual in the breeding scheme based on the estimated genomic marker (i.e. SNPs) effects throughout the genome^23^. The individuals carrying the most extreme GEBVs within the two lines, produced the F2 generation. Of the F2, 36 breeding pairs (late selection line n = 18, early selection line n = 18) were housed in climate-controlled aviaries from January until July and subjected to contrasting temperature environments, mimicking a cold (2013) or warm (2014) spring in The Netherlands. Birds were allowed to breed and their egg laying date (the date at which they laid their first egg) was recorded.

**Fig. 1:**
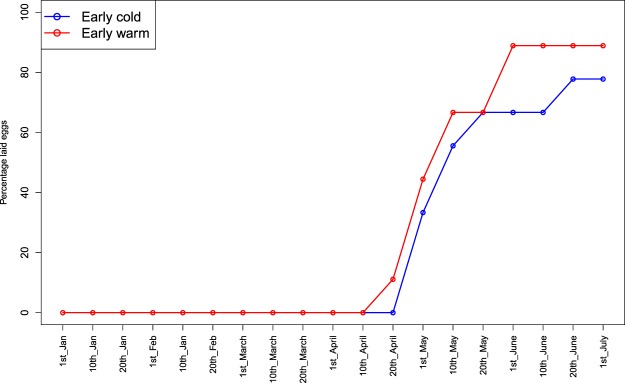
An illustrated figure in which the percentage of females laying within the warm (red) and cold (blue) treatment are calculated per blood sampling moment. Females from a selection line for early reproduction were exposed to two different temperature regimes warm (red) or cold (blue) housed in climate-controlled aviaries from January until end of July. Blood samples were collected biweekly from each individual during this time. Egg laying was monitored daily from mid-March onwards.

Blood samples were collected from the birds every other week during the experiment (from 28-01 until 07-07-2016), with half the birds sampled in odd weeks and the other half sampled in even weeks (Fig. 1). For every sampling moment, all four line × treatment combinations were represented. After weighing, a blood sample (max. 150 µl) was taken from the jugular vein with a syringe (Easy Touch Insulin, 0.3 ml with 31 G). All birds were sampled within 10 minutes of capture. Plasma was separated from red blood cells with a Hamilton syringe after centrifuging at 14000 rpm for 10 minutes and the red blood cells were stored in Queens buffer (0.01 M Tris, 0.01 M NaCl, 0.01 M EDTA, 1% n-lauroylsarcosine, pH 8.0)^24^ at room temperature until being processed. The experiment was performed under the approval by the Animal Experimentation Committee of the Royal Academy of Sciences (DEC-KNAW), Amsterdam, The Netherlands, protocol NIOO 14.10.

For this study, we used the red blood cell samples from the early selection line females (n = 16) from both temperature treatments. In addition, four sampling time points were chosen for analysis based on the known effects of photoperiod on reproduction^19^ and the realized lay dates of the individuals; (1) the day when day light length >12hrs (time point 1), (2) the day when 25% of the females from the warm environment had initiated laying (time point 2), (3) the day when 25% and 50% of the females from the cold and warm environment respectively had initiated laying (time point 3) and (4) the day when 50% of the females from the cold treatment had initiated laying. As these time points do not coincide with the days of blood sampling we chose blood samples closest to (+/−7 days) the four time points. One female (warm treatment, time point 4) was incubating at the time so we could not take a blood sample at this stage. The total number of samples is therefore 63. Because of the blood sampling scheme and that females were incubating eggs (and hence were not sampled) there is a one- to two-week difference between the exact sampling days within a time point (Fig. 1).

### Data analysis

**Fig. 2:**
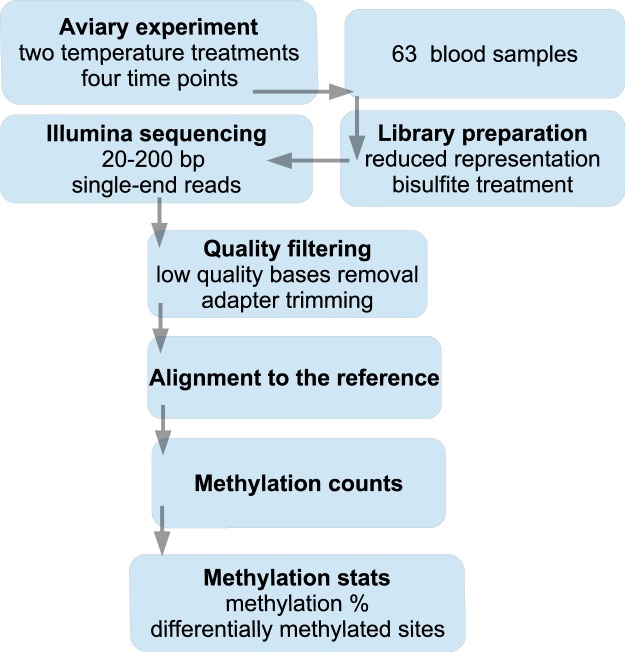
A schematic overview of the work flow to generate the data. First great tit offspring were collected from the wild were used to generate F1 and F2 generations for the aviary experiment. Blood samples from F2 females were collected from four time points and two thermal treatments. Standard Illumina protocols were used for library preparation and sequencing. Several quality control steps were performed for raw sequencing reads for subsequent filtering steps. Quality filtered reads were mapped against the reference sequence and methylation counts were recorded for statistical analyses.

The overall work flow of this study is described in Fig. 2. Total genomic DNA was extracted using FavorPrepT M 96-well Genomic DNA Kit (Favorgen). RNA was removed with an RNAse treatment. DNA Quality and quantity was assessed using a Nanodrop 2000 (Agilent Biotechnologies) and by 1% agarose gel electrophoresis. Approximately 1 ug of total genomic DNA was used for library preparation. A reduced representation library preparation protocol was used according to manufacturer’s protocol (Illumina). DNA samples were first digested with restriction enzyme MspI to generate CCGG overhangs. Fragmented DNA was then bisulfite treated, which converts un-methylated cytosine nucleotides to thymine nucleotides. Fragmented and bi-sulfite treated DNA was then end-repaired with DNA polymerase I and A-overhangs were added to the 3’ ends of each fragment for adapter ligation. Standard Illumina adapters containing individual barcodes were used for identification of sequencing reads in the downstream analyses after the sequencing. The libraries were size-selected for fragment sizes 20–200 basepairs (bp), and concentrations were determined by quantitative PCR. Sixteen libraries were pooled into the same sequencing lane of a flow cell by randomizing individuals, sampling days and treatments to avoid lane effects^25^. Altogether eight lanes were used for sequencing such that each pooled set was sequenced on two lanes with 100 bp from single end reads. All the pools were run on the same flow cell on a HiSeq. 2500 sequencer using a HiSeq SBS sequencing kit version 4 (Illumina). An internal positive control (PhiX) was used to obtain reliable sequence generation in the sequencing processing. Library preparation and sequencing were performed at the Roy J. Carver Biotechnology Center, University of Illinois at Urbana-Champaign, USA.

**Fig. 3:**
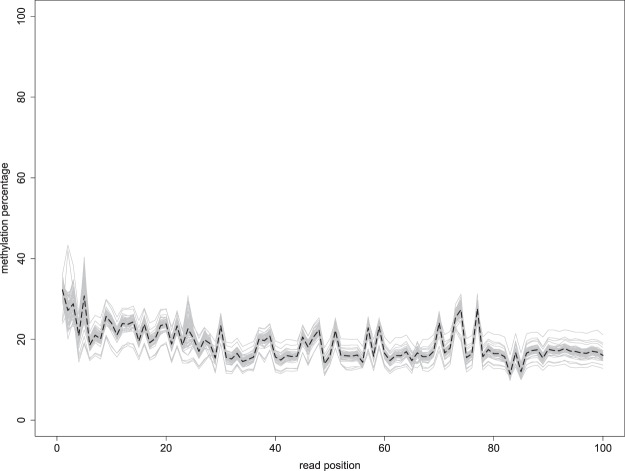
Methylation bias plot in CpG context. The grey line shows the mean methylation percentage across all sequence reads in one sequence library in each position of the sequence read. The black dashed line shows the mean methylation percentage across all libraries.

The quality of the sequencing reads was investigated as implemented in the FastQC 0.11.2 quality control tool^25,26^. The quality control analysis indicated presence of low quality bases in the 3’ end of the reads^25^. The low quality bases and adapter contamination were trimmed using Trim Galore! 0.4.2^27^ with default parameters^25^. In order to obtain methylation counts sequencing reads were aligned against the Great tit reference genome v1.1^28^ using Bismark 0.16.3 aligner^29^. Sites in sequence reads containing Cs in comparison to reference sequence were taken as methylated sites whereas Ts were taken as un-methylated sites. The estimation of methylation percentage was based on the relative proportion of methylated and un-methylated sites. The methylation bias (M-bias)^30^, i.e. if the methylation level at different position of the read varies, was examined by plotting the average methylation percentage in each position along the read in CpG context (Fig. 3). As can be seen from Fig. 3 there is relatively little technical variation in methylation across the reads, although, depending on the application of these data, the first 5 bp should possibly be removed, as methylation levels are a bit higher in this region.

Estimation of bisulphite conversion rate was based on proportion of C/T in the mitochondria to the reference genome. As mitochondria is mostly un-methylated it can be used to estimate conversion efficiency by comparing the amount of C/T in the reference sequence to what was acquired by RRBS^31^. Similarly, non-CpG methylation is negligible in great tit red blood cells^28,32^ and these sites can also be used to estimate bisulphite conversion efficiency. Using mitochondria, the conversion efficiency is 99.8% and based on non-CpG methylation it is 98.6%^25^.

We obtained on average 20.16 million raw sequencing reads per library, of which 20.05 million remained after quality filtering and with average coverage of 13.6x per CpG site per sample^25^. As reads were of good quality prior to trimming, read lengths after trimming were hardly affected^25^. Of these trimmed reads, 10.50 million quality-filtered reads were mapped uniquely to the great tit reference genome^25^, resulting in average mapping efficiency of 52.0 ± 2.0%. There are several reasons that could explain the rather large proportion of unmapped reads. First, unmapped reads can be contamination from another organism during e.g. library preparation or field sampling. To examine this, we took a subset of first 5,000 unmapped reads from four sequencing libraries and we used Blast search against the non-redundant nucleotide database available at GenBank. The majority of the unmapped reads did not have a Blast hit indicating that contamination of our libraries was unlikely (Table 1). Second, read mapping could be compromised due to incomplete reference genome. The published great tit genome contains a large number (~ 1,500) unordered scaffolds, indicating that some parts of the genome are not included in the assembly in their correct genomic location although they are included in the reference used in the alignment. Thus, it is possible that reads do not perfectly align to the scaffolds, especially at the ends of them. Also, while mapping efficiency seems low it is comparable to other methylation studies in great tits. For example, Derks *et al*.^32^ used whole genome methylation sequencing and found that 52% (brain) and 64% (blood) of the reads were mapped against the same great tit reference genome as used in this study. We did however observe a large proportion of very short (about 30 bp) reads among the unmapped reads which are known to be challenging to map against any reference for the current alignment software^33^ and thus might explain some of the reason for the relatively low mapping success.

**Table 1:** Results of the Blast searches of unmapped reads from four libraries.

library	no hit	aves	mammalia	other
BD 27012_1	4759	10	6	255
BD 27012_2	4957	8	6	29
BD 27012_3	4955	8	6	31
BD 27012_4	4952	12	1	35

Altogether 11,057,686 methylated sites across 63 samples were identified for differential methylation analyses^25^. These sites covered 71.9% of all known CpG sites in the great tit genome. The mean methylation level was 21.54 ± 1.45%^25^, which is similar to that observed in a single male individual using whole-genome bisulfite sequencing^28^ and in another RRBS dataset on great tits^32^. Non-CpG methylation in the samples was low (0.46 ± 0.15%, mean and sd)^25^. The identified CpG sites covered 80% of the genes in the current great tit annotation (version 1.1) and encompassed different genomic locations, estimated using the R packages GenomicFeatures^34^ and rtracklayer^35^. Identified sites were annotated to introns (39.9%), exons (34.3%), promoters (10.3%) and intergenic regions (15.4%). From earlier work on the great tit, we know that gene expression is associated differentially with these regions^32,36^. Depending on total coverage cut-off, numbers of sites shared across all samples drop quite quickly (Table 2.). Mean methylation level in included and excluded sites also drops when requiring higher total coverage per site and the site to present with required coverage in all 16 samples and all 4 sampling time points. This is mainly because of two things: (1) when filtering for coverage across all samples we are excluding single sites which have high methylation level but which are present in some individuals only and (2) estimation of methylation level is based on low total coverage and can thus be erroneous. Thus, we encourage the use of total coverage when further filtering the data set for downstream analysis to allow more accurate calling of methylation level for any type of downstream analysis.

**Table 2:** Numbers of sites identified across all samples with different total coverage cut-offs (no cut off, 1x, 3x, 5x and 10x) and methylation levels in the included and excluded sites. Coverage cut off was required for all 16 individuals across the 4 time points.

	No coverage filtering	1x	3x	5x	10x
CpG sites	11 057 685	2 653 390	2 217 299	1 730 250	522 645
Methylation % selected	21.54	18.8	17.45	16.45	13.82
Methylation % excluded			25.65	23.2	20.02

The temporally replicated DNA methylation dataset reported here is one of very few genome-wide characterizations of DNA methylation in ecological model species available and will serve as an important resource for future studies examining the stability and repeatability of methylation and the link between methylation and timing of reproduction. It will also be an important resource for future comparative studies of DNA methylation patterns in birds.

## Data Records

The data described here consists of sequence reads deposited in NCBI Sequence Read Archive^37^. All the libraries and the individual fastq files contained in them are deposited under accessions SRX3209916-SRX3209919^37–40^. The Figshare records^25^ comprise of four files. First file is a summary table including information on sequencing design, mapping statistics and methylation levels in different contexts. Second and third file show sequencing quality reports before and after quality trimming, respectively. The fourth is a table reporting raw methylation counts and methylation level for each CpG site.

## Technical Validation

**Fig. 4:**
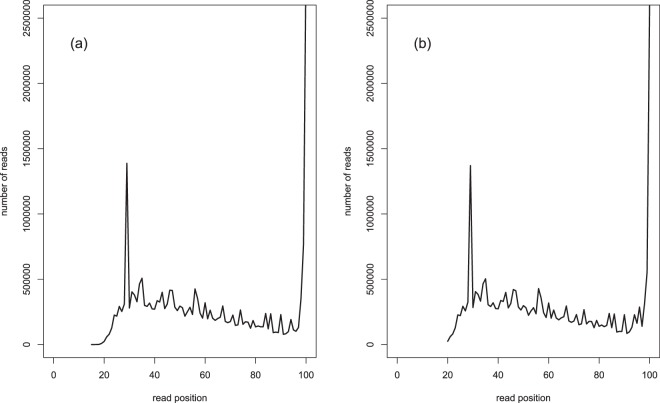
Representative plots of read length distributions. (**a**) Shows the read length distribution in a raw sequence library. (**b**) Shows the read length distribution in a filtered sequence library. On the X axis is the read length and on the Y axis is number of occurrences of reads of specified lengths.

Quality filtering steps taken to ensure the sequences^25^ are of good quality are described in Methods section. The effect of quality filtering is presented in^25^ with counts of the raw reads as well as reads after trimming for low quality bases at the 3’ end^25^ and length distributions of raw reads and after trimming (Fig. 4). We acknowledge the potential presence of PCR duplicates in the dataset resulting in low sequence complexity. As most of the reads in the data will have identical start-stop coordinates as a result of RRBS library preparation, deduplication based on just coordinates is not recommended^41^. Finally, masking the genome for G/C polymorphisms might lead to more accurate calling of methylated loci^41^. Such effect has been observed in humans when inter-population divergence was taken into account^42^. However, the samples in this study originate from the same natural population and we expect that the effect of background polymorphisms on the identification methylation calling is not greatly compromised.

## ISA-Tab metadata file

Download metadata file
